# Long-term evaluation of the timing of corticosteroid therapy in an IgA nephropathy cohort

**DOI:** 10.1093/ckj/sfaf076

**Published:** 2025-03-17

**Authors:** Wanyin Hou, Haiyan Yang, Pei Chen, Chen Tang, Xujie Zhou, Lijun Liu, Li Zhu, Sufang Shi, Jicheng Lv, Hong Zhang

**Affiliations:** Renal Division, Department of Medicine, Peking University First Hospital, Institute of Nephrology, Peking University, Key Laboratory of Renal Disease, Ministry of Health of China, Beijing, China; Department of Nephrology and Rheumatology, Zunyi Hospital of Traditional Chinese Medicine, Zunyi, Guizhou, China; Renal Division, Department of Medicine, Peking University First Hospital, Institute of Nephrology, Peking University, Key Laboratory of Renal Disease, Ministry of Health of China, Beijing, China; Renal Division, Department of Medicine, Peking University First Hospital, Institute of Nephrology, Peking University, Key Laboratory of Renal Disease, Ministry of Health of China, Beijing, China; Renal Division, Department of Medicine, Peking University First Hospital, Institute of Nephrology, Peking University, Key Laboratory of Renal Disease, Ministry of Health of China, Beijing, China; Renal Division, Department of Medicine, Peking University First Hospital, Institute of Nephrology, Peking University, Key Laboratory of Renal Disease, Ministry of Health of China, Beijing, China; Renal Division, Department of Medicine, Peking University First Hospital, Institute of Nephrology, Peking University, Key Laboratory of Renal Disease, Ministry of Health of China, Beijing, China; Renal Division, Department of Medicine, Peking University First Hospital, Institute of Nephrology, Peking University, Key Laboratory of Renal Disease, Ministry of Health of China, Beijing, China; Renal Division, Department of Medicine, Peking University First Hospital, Institute of Nephrology, Peking University, Key Laboratory of Renal Disease, Ministry of Health of China, Beijing, China; Renal Division, Department of Medicine, Peking University First Hospital, Institute of Nephrology, Peking University, Key Laboratory of Renal Disease, Ministry of Health of China, Beijing, China

**Keywords:** corticosteroid, IgA nephropathy, timing, treatment

## Abstract

**Background:**

Current proposed KDIGO guidelines suggest systemic corticosteroid therapy to reduce glomerular inflammation in immunoglobulin A nephropathy (IgAN), however the optimal timing for initiating steroid treatment remains a topic of debate. This study evaluates the impact of early versus delayed steroid initiation on long-term outcomes in IgAN patients.

**Methods:**

We conducted a retrospective study of 268 IgAN patients treated with corticosteroids for >3 months within 3 years of kidney biopsy. Patients were categorized into early therapy (steroids within 30 days) and delayed therapy (after 30 days). Propensity score matching created matched cohorts. Kaplan–Meier curves and Cox regression assessed outcomes. The primary endpoint was a composite renal outcome [estimated glomerular filtration rate (eGFR) >50% reduction, end-stage kidney disease or renal death]. Secondary endpoints included eGFR decline >30% or >40% and an eGFR slope and time-average proteinuria.

**Results:**

Propensity score matching identified 191 individuals for analysis. The primary outcome was significantly better in the early therapy group, with a hazard ratio (HR) of 0.41 [95% confidence interval (CI) 0.17–0.96, *P* = .041]. Significant benefits were also observed for secondary outcomes, including a lower frequency of >30% and >40% eGFR decline in the early therapy group, with HRs of 0.48 (95% CI 0.24–0.98, *P* = .04) and 0.34 (95% CI 0.14–0.81, *P* = .01), respectively. Cox regression confirmed that the timing of steroid initiation (early vs delayed) was a significant factor associated with kidney progression [HR = 0.33 (95% CI 0.14–0.77), *P* = .01]. The average eGFR slope over 10 years was more favorable in the early therapy group (–1.0 ± 6.0 vs –2.9 ± 6.8 mL/min/1.73 m^2^ per year, *P* = .039). No significant differences in baseline characteristics were found to influence the timing of steroid use in progressive IgAN.

**Conclusions:**

Early corticosteroid therapy may help reduce renal decline and preserve long-term kidney function in IgAN patients requiring steroid treatment.

KEY LEARNING POINTS **What was known:**

- Current KDIGO guidelines suggest systematic corticosteroid therapy to reduce glomerular inflammation in IgAN; however, the optimal timing for initiating steroid treatment remains a topic of debate.

**This study adds:**

- Our results indicate early corticosteroid therapy may help reduce renal decline and preserve long-term kidney function in IgAN patients.

**Potential impact:**

- Our study offers critical insights into the optimal timing for steroid therapy in IgAN.
- These results may have important implications for clinical practice and the management of progressive IgAN, particularly in terms of refining treatment strategies and improving patient prognosis.

## INTRODUCTION

Immunoglobulin A nephropathy (IgAN) is the most common primary glomerulonephritis in the Chinese population, accounting for more than 50% of primary glomerulonephritis diagnosed by kidney biopsy [1]. It is characterized by the deposition of IgA in the glomerular mesangial on kidney biopsy, representing a poor prognosis and leading cause of end-stage kidney disease (ESKD). Long-term outcomes are generally unfavorable, with few patients expected to avoid kidney failure over their lifetime [2]. The pathogenesis of IgAN is widely described by the “four-hit” hypothesis, which associates aberrant glycosylation of IgA1 (Gd-IgA1) with the formation of immune complexes. These complexes deposit in the renal mesangium, triggering an inflammatory response [3]. Thus, IgAN is recognized as autoimmune kidney disease, supporting the rationale for therapeutic strategies involving corticosteroids and immunosuppressive agents to modulate the immune response and slow disease progression [4].

The use of systemic glucocorticoids in the treatment of IgAN has long been debated, particularly regarding their indication, optimal dosage and timing. Randomized controlled trials like the TESTING (Therapeutic Evaluation of Steroids in IgA Nephropathy Global) [5, 6] and STOP-IgAN (Supportive Versus Immunosuppressive Therapy for the Treatment of Progressive IgA Nephropathy) [7] trials have provided valuable but conflicting results. The TESTING trial showed that steroids reduced renal failure risk in high-risk IgAN patients with more severe proteinuria and impaired renal function, though this benefit was accompanied by a higher incidence of adverse events.

Current proposed Kidney Disease: Improving Global Outcomes (KDIGO) guidelines suggest systemic corticosteroids therapy for reduce glomerular inflammatory in IgAN [8]. While the optimal timing for initiating steroid treatment remains a topic of debate. Limited studies have specifically addressed whether corticosteroids should be administered early, immediately following diagnosis, or reserved for patients unresponsive to renin–angiotensin system inhibitors and other supportive treatments. Some advocate for early corticosteroid intervention to prevent irreversible kidney damage, while others recommend delaying immunosuppressive therapy until supportive care options are fully exhausted to minimize steroid-related complications. These uncertainties highlight the pressing need for further research to refine treatment protocols for IgAN, particularly in the context of timing and patient selection.

The treatment landscape for IgAN has evolved significantly with the development of novel therapies targeting the underlying disease mechanisms, particularly those involving IgA molecules and immune complexes [9–13]. These emerging therapeutic options have expanded the range of treatments available for IgAN, but their introduction has also made the use of steroids in managing the disease more complex. According to the 2024 KDIGO guidelines, despite well-established concerns regarding their side effects, steroids and other immunosuppressive agents remain central to the management of progressive IgAN, especially in regions where access to newer, more expensive therapies is limited. More importantly, the guidelines suggest that the treatment strategy for most patients with IgAN should simultaneously include reducing pathogenic IgA immune complex formation, decreasing inflammation and providing supportive therapy [8].

In light of these advancements, the management of IgAN now requires a more refined approach, with treatment decisions guided by a comprehensive assessment of the patient's condition, including proteinuria, renal function and renal pathology [14]. The timing of steroid initiation has emerged as a critical factor influencing long-term outcomes in IgAN.

The KDIGO guidelines underscore that the risk of adverse events associated with steroid therapy is higher in patients with an estimated glomerular filtration rate (eGFR) <50 mL/min/1.73 m^2^ [15], highlighting the importance of careful patient selection. Given these considerations, the present study seeks to evaluate the impact of early versus delayed steroid initiation on the long-term prognosis of IgAN.

## MATERIALS AND METHODS

### Population

We conducted a retrospective study to investigate the timing of steroid use in patients with IgAN. A total of 1995 patients with complete follow-up data, treated between 1996 and 2021, were screened at Peking University First Hospital. The inclusion criteria were: (i) primary IgAN confirmed by kidney biopsy; (ii) eGFR ≥50 mL/min/1.73 m^2^ at the time of renal biopsy; (iii) corticosteroid therapy initiated within 14 days before or up to 3 years after kidney biopsy. Corticosteroid therapy was defined as follows: (i) an initial dose of steroid or (prednisone ≥30 mg/day, methylprednisolone ≥24 mg/day) and (ii) therapy lasting for at least 3 months. (iii) All included patients received standard therapy, including angiotensin-converting enzyme inhibitors (ACEi) or angiotensin-receptor blockers (ARB) to reduce hyperfiltration, blood pressure control and lifestyle modifications, prior to initiating corticosteroid treatment. The exclusion criteria included: (i) secondary IgAN, such as lupus nephritis, cirrhosis, Henoch-Schönlein purpura nephritis or infection-related nephropathy; (ii) patients presenting with nephrotic syndrome and renal pathology indicative of minimal change disease; (iii) kidney biopsy samples containing fewer than eight glomeruli; and (iv) missing follow-up data on corticosteroid therapy. We define the corticosteroid treatment period as within 3 years based on findings from the STOP-IgAN study, which reported no significant impact of corticosteroid therapy on the rate of eGFR decline or progression to ESKD over this timeframe [7]. The eGFR was calculated using the creatinine equation of the Chronic Kidney Disease Epidemiology Collaboration (CKD-EPI) [16].

This study was conducted according to the Declaration of Helsinki. All subjects provided informed consent to participate in this study, and this study is approved by Peking University First Hospital Ethics Committee [approval number 2003(0302), 2013(548), 2020(159)].

### Study design

This is a retrospective observational study. Patients were divided into two groups based on the time from kidney biopsy to the start of corticosteroid therapy: within 30 days (early therapy) and after 30 days group (delayed therapy). We analyze the timing of corticosteroid use in relation to primary endpoint and secondary endpoint. The primary endpoint includes a composite of renal events, defined as a reduction in eGFR of more than 50%, ESKD or renal death. The secondary endpoints include a ≥30% and ≥40% reduction in eGFR, the eGFR slope and the time-average proteinuria within 1 year following corticosteroid treatment in each group.

### Statistical analysis

The statistical distribution of the study data was described as mean ± standard deviation (SD) and compared using independent samples *t*-tests or paired samples *t*-tests. Non-parametric variables were described using the median and interquartile range (IQR) and compared using the Mann–Whitney U test. To determine the optimal timing for immunosuppressive therapy in IgAN patients, we employed Kaplan–Meier survival curves and multifactorial Cox regression analyses to compare differences in the occurrence of composite endpoint events. All *P*-values were two-tailed, and a value of <.05 was considered statistically significant. We performed propensity score matching (delayed vs early therapy 1:2) based on age, gender, mean arterial pressure (MAP), serum creatinine, eGFR, 24-h urine proteinuria and the Oxford Classification (MEST-C) scores before kidney biopsy. The nearest-neighbor matching algorithm without replacement was employed with a caliper width of 0.2 SDs of the logit of the propensity score to ensure close matches. To ensure valid comparisons between the two groups, baseline clinical and pathological characteristics were balanced, with no significant differences observed between them following matching. Confidence intervals (CIs) included 95% predictive values. Statistical analyses were conducted using SPSS software version 22.0 (IBM Corp., Armonk, NY, USA) and R programming software version 3.4.1.

## RESULTS

### Baseline characteristics

**Figure 1:**
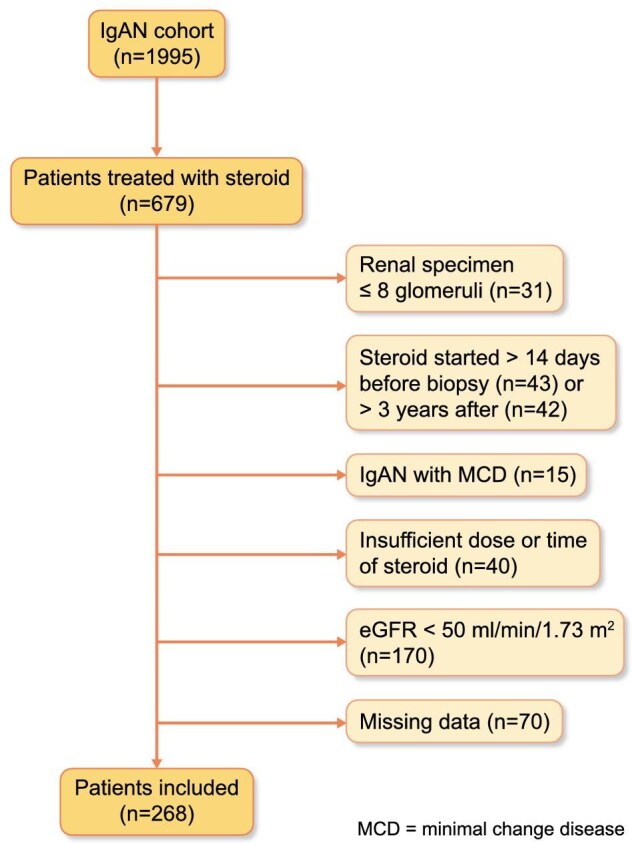
The process of patient screening.

A total of 268 eligible patients with IgAN receiving steroid therapy were enrolled in this study. The screening process is illustrated in Fig. 1. The median follow-up duration was 59 months (IQR 34–99 months). Of the cohort, 137 (51.1%) were male and 131 (48.9%) were female. Baseline clinical characteristics included a median proteinuria level of 2.3 g/day (IQR 1.0–4.2 g/day), a MAP of 93 ± 11 mmHg and an average GFR of 87 ± 24 mL/min/1.73 m^2^. According to the Oxford Classification, the distribution of lesions was as follows: M0/M1 (61.9%/38.1%), E0/E1 (55.2%/44.8%), S0/S1 (36.2%/63.8%), T0/T1/T2 (69.0%/24.3%/6.7%) and C0/C1/C2 (30.6%/44.4%/25.0%). In terms of treatment initiation, 189 patients (70.5%) received steroids therapy within 30 days of diagnosis, 26 patients (9.8%) started steroids therapy between 30 and 90 days, and 53 patients (19.7%) began steroids therapy more than 90 days after diagnosis. We divided the IgAN patients into two groups based on the timing of steroid initiation: those who started treatment within 30 days (early therapy) and those who began after 30 days (delayed therapy). As shown in Table 1, early therapy had a significantly higher proportion of female patients compared with the after 30 days group (male/female ratio: 0.89 vs 1.54, *P* = .041). Moreover, patients in the early therapy had significantly more C2 lesions, while the delayed therapy group had a higher proportion of C1 lesions (C0/C1/C2 in the within vs after 30 days: 30.2%, 38.1%, 31.7% vs 31.6%, 59.5%, 8.9%, *P* < .001). To compare the prognosis between the two groups, 191 IgAN patients were included after propensity score matching, which controlled for baseline characteristics such as age, gender, MAP, serum creatinine, eGFR, 24-h urine proteinuria and the Oxford Classification (MEST-C) scores, ensuring that clinical characteristics were well balanced.

**Table 1:** Demographic characteristics of enrolled individuals and included patients by propensity score.

	Unmatched (*n* = 268)			Matched (*n* = 191)		
Basiline characteristics	Early therapy group (*n* = 189)	Delayed therapy group (*n* = 79)	*P*	Early therapy group (*n* = 113)	Delayed therapy group (*n* = 78)	*P*
Gender, *n* (%)			.041			.999
Male	89 (47.1)	48 (60.8)		67 (59.3)	47 (60.3)	
Female	100 (52.9)	31 (39.2)		46 (40.7)	31 (39.7)	
Age (years)	33 ± 13	35 ± 12	.239	35 ± 13	35 ± 13	.912
History of diabetes, *n* (%)	4 (2.1)	3 (3.8)	.431	2 (1.8)	3 (3.8)	.377
MAP (mmHg)	93 ± 11	93 ± 11	.810	93 ± 10	93 ± 11	.916
eGFR (mL/min/1.73 m^2^)	88 ± 25	86 ± 24	.692	87 ± 23	86 ± 24	.671
UTP (g/24 h)	2.5 (1.2–4.5)	1.5 (0.8–4.0)	.016	2.2 (0.9–3.8)	1.6 (0.8–4.0)	.293
Hematuria (/μL)	15 (4–81)	21 (6–92)	.321	14 (5–78)	17 (4–86)	.551
Oxford Classification, *n* (%)						
M			.278			.752
M0	121 (64.0)	45 (57.0)		69 (61.1)	45 (57.7)	
M0	68 (36.0)	34 (43.0)		44 (38.9)	33 (42.3)	
E			.047			.446
E0	97 (51.3)	51 (64.6)		65 (57.5)	50 (64.1)	
E1	92 (48.7)	28 (35.4)		48 (42.5)	28 (35.9)	
S			.470			.653
S0	71 (37.6)	26 (32.9)		41 (36.3)	25 (32.1)	
S1	118 (62.4)	53 (67.1)		72 (63.7)	53 (67.9)	
T			.400			.893
T0	127 (67.2)	58 (73.4)		79 (69.9)	57 (73.1)	
T1	47 (24.9)	18 (22.8)		29 (25.7)	18 (23.1)	
T2	15 ( 7.9)	3 ( 3.8)				
C			<.001			.778
C0	57 (30.2)	25 (31.6)		37 (32.7)	24 (30.8)	
C1	72 (38.1)	47 (59.5)		63 (55.8)	47 (60.3)	
C2	60 (31.7)	7 ( 8.9)		13 (11.5)	7 ( 9.0)	

UTP, 24-h urinary total protein quantity.

### Corticosteroid timing and IgAN outcomes

The primary composite outcome, defined as a decline in eGFR of at least 50%, ESKD or death from renal causes, occurred in 30 participants (15.9%) in the early therapy group and in 18 participants (22.8%) in the delayed therapy group. For the secondary endpoint, 45 patients (23.8%) in the early therapy group had a reduction in eGFR of >30%, while 35 patients (18.5%) had a reduction of >40%. In the delayed therapy group, 24 patients (30.4%) exhibited a reduction in eGFR >30%, and 18 patients (22.8%) had a reduction of >40%. For the entire population before propensity score matching, Kaplan–Meier survival analysis showed no significant difference between the early therapy group and the delayed therapy group in primary outcomes (Log rank *P *= .21) (Supplementary data, Fig. S1).

After propensity score matching, the event rates for the components of the composite outcome over 10 years are summarized in Table 2. The proportion of participants with the primary composite outcome was significantly lower in the early therapy group compared with those in the delayed therapy group, with a hazard ratio (HR) of 0.41 (95% CI 0.17–0.96, *P* = .041). For the secondary endpoints, a significant difference was observed in the proportion of participants with a decline in eGFR ≥30% or ≥40% between the two groups. Specifically, the early therapy group had a lower risk of a 30% decline in eGFR, with a HR of 0.48 (95% CI 0.24–0.98, *P* = .04), and a HR of 0.34 (95% CI 0.14–0.81, *P* = .01) for a 40% decline in eGFR, compared with the delayed therapy group.

**Table 2:** Primary and secondary outcomes for different timing of steroid.

	Early therapy (*n* = 113)	Delayed therapy (*n* = 78)	
Outcome	*n*/total *n* (%)	*n*/total *n* (%)	HR (95% CI), *P* for difference
Primary composite outcome	10/113 (8.8)	15/78 (19.2)	0.41 (0.17–0.96), 0.041
Decline in eGFR ≥50%	7/113 (6.2)	14/78 (17.9)	0.30 (0.12–0.79), 0.014
Renal replacement therapy	4/113 (3.5)	7/78 (9.0)	0.37 (0.11–1.32), 0.126
eGFR <15 mL/min/1.73 m^2^	2/113 (1.8)	5/78 (6.4)	0.26 (0.05–1.39), 0.116
Death from renal causes	1/113 (0.9)	0/78 (0)	
Secondary outcome			
Decline in eGFR ≥30%	18/113 (15.9)	22/78 (28.2)	0.48 (0.24–0.98), 0.043
Decline in eGFR ≥40%	9/113 (8.0)	16/78 (20.5)	0.34 (0.14–0.81), 0.014

**Figure 2:**
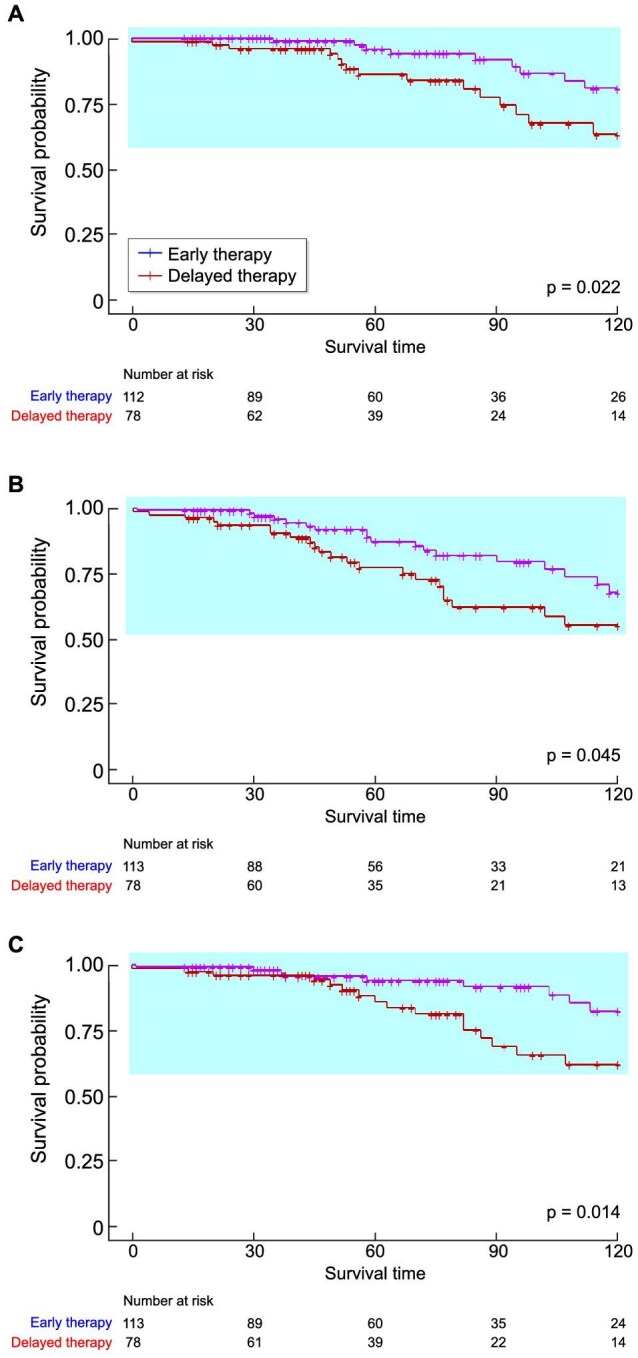
Kaplan–Meier survival analysis. (**a**) Primary endpoint, (**b**) ≥30% decline in eGFR and (**c**) ≥40% decline in eGFR.

Kaplan–Meier survival analysis demonstrated a significant difference in the occurrence of the composite endpoint between the two groups, indicating superior renal survival in the early therapy group (Log-rank *P* = .022). Similarly, the occurrence of a 30% or 40% decline in eGFR also differed significantly between the groups, with Log-rank *P*-values of .045 and .014, respectively (Fig. 2). We then conducted a Cox regression analysis to assess the differences in the occurrence of composite endpoint events (Table 3). In the univariate analysis, delayed initiation of steroid therapy and the presence of Oxford T lesions were significantly associated with composite events in patients with IgAN. After adjusting for factors such as age, gender, 24-h urinary protein, MAP and MEST-C classification, early initiation of therapy was identified as a protective factor against composite endpoint events, with an HR of 0.33 (95% CI 0.14–0.77, *P* = .01).

**Table 3:** Cox regression analysis of factors Associated with composite renal outcomes.

	Univariate analysis		Multivariate analysis^a^	
	HR (95%)	*P-*value	HR (95%)	*P*-value
Group				
Delayed therapy	Reference		Reference	
Early therapy	0.44 (0.20, 0.97)	.043	0.33 (0.14, 0.77)	.010
Age (years)	0.99 (0.95, 1.02)	.392		
Gender, male/female	1.62 (0.68, 3.88)	.281		
eGFR, CKD EPI (mL/min/1.73 m^2^)	0.99 (0.97, 1.01)	.213		
Proteinuria UTP (g/day)	1.01 (0.86, 1.19)	.909		
MAP	1.02 (0.98, 1.06)	.447		
Oxford Classification				
Mesangial hypercellularity				
M0/M1	1.38 (0.63, 3.03)	.421		
Endocapillary hypercellularity				
E0/E1	0.80 (0.33, 1.92)	.614		
Segmental glomerulosclerosis				
S0/S1	1.91 (0.71, 5.09)	.198		
Tubular atrophy/interstitial fibrosis				
T0/1	3.94 (1.73, 9.00)	.001	5.26 (1.93,14.39)	.001
T1/2	2.63 (0.57, 12.00)	.213	2.59 (0.43, 15.49)	.297
Crescents				
C0/1	1.38 (0.54, 3.49)	.503		
C1/2	1.05 (0.21, 5.22)	.951		

*P *< .05 was considered significant.

a Multivariate model: all clinical and pathologic parameters significantly associated with renal outcome were included.

UTP, 24-h urinary total protein quantity.

### Timing of corticosteroid therapy on eGFR and proteinuria

**Figure 3:**
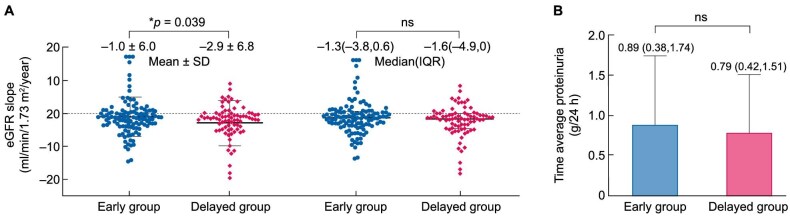
Timing of corticosteroid therapy on eGFR and proteinuria. (**a**) The first two columns represent the *P*-value for the comparison of average eGFR slopes using the independent samples *t*-test, while the last two columns show the *P*-value obtained from nonparametric tests. (**b**) The columns indicate that there is no difference in the time-averaged proteinuria within 1 year after corticosteroid therapy between the early and delayed therapy groups.

To assess the impact of early versus delayed initiation of steroid therapy on the 10-year prognosis of IgAN, we further compared the eGFR slopes between the two groups. As shown in Fig. 3a the average eGFR slope in the early therapy group was –1.0 mL/min/1.73 m^2^ per year, compared with –2.9 mL/min/1.73 m^2^ per year in the delayed therapy group (*P* = .039). However, when using the median values, the difference was not significant: –1.3 (–3.8, 0.6) vs –1.6 (–4.9, 0) mL/min/1.73 m^2^ per year (*P* = .294). We then explored changes in time average proteinuria over the course of 1 year following corticosteroid treatment in the two groups. As shown in Fig. 3b, there was no significant difference in time average proteinuria between the early and delayed corticosteroid treatment groups.

### Survival outcomes in patients with IgAN

To investigate the impact of the timing of steroid therapy initiation on renal survival, Table 4 summarizes the renal survival rates over different time points. Renal survival is defined as a decline in eGFR <30% (Survival 1), <40% (Survival 2) or <50% (Survival 3). The data includes follow-up at 1-year, 3-year, 5-year, 7-year and 9-year intervals. The median follow-up duration was 64 months (IQR 32–113) in the early therapy group, compared with 62 months (IQR 40–100) in the delayed therapy group (*P* = .857). Overall, the early therapy group demonstrated a higher renal survival rate compared with the delayed therapy group. These differences became statistically significant at the 7-year follow-up for renal survival based on eGFR declines of <30% (79.9% vs 62.3%, *P* = .044) and <50% (93.2% vs 81.9%, *P* = .028). At the 9-year follow-up, renal survival (eGFR decline <50%) remained significantly higher in the early therapy group (87.6% vs 68.5%, *P* = .028) than in the delayed group.

**Table 4:** Renal survival during follow-up in different timing of steroid therapy.

	Early therapy (*n* = 113)			Delayed therapy (*n* = 78)					
Time (years)	Survival 1, *n* (%)	Survival 2, *n* (%)	Survival 3, *n* (%)	Survival 1, *n* (%)	Survival 2, *n* (%)	Survival 3, *n* (%)	*P* ^1^	*P* ^2^	*P* ^3^
1	112 (99.1)	112 (99.1)	112 (99.1)	76 (97.4)	77 (98.7)	77 (98.7)	.359	.791	.791
3	76 (95.7)	78 (96.7)	78 (98.0)	57 (90.3)	60 (96.0)	61 (97.3)	.183	.465	.808
5	54 (87.2)	59 (94.0)	58 (94.9)	35 (77.2)	38 (85.8)	39 (87.4)	.095	.125	.136
7	37 (79.9)	39 (88.4)	41 (93.2)	22 (62.3)	25 (75.2)	26 (81.9)	.044	.100	.028
9	25 (71.8)	27 (80.7)	29 (87.6)	14 (55.3)	16 (62.0)	16 (68.5)	.07	.07	.028

Survival 1: decline in eGFR by <30%, *P*^1^ for difference between within 30 days and after 30 days in Survival 1.

Survival 2: decline in eGFR by <40%, *P*^2^ for difference between within 30 days and after 30 days in Survival 2.

Survival 3: decline in eGFR by <50%, *P*^3^ for difference between within 30 days and after 30 days in Survival 3.

### Subgroup analyses

**Figure 4:**
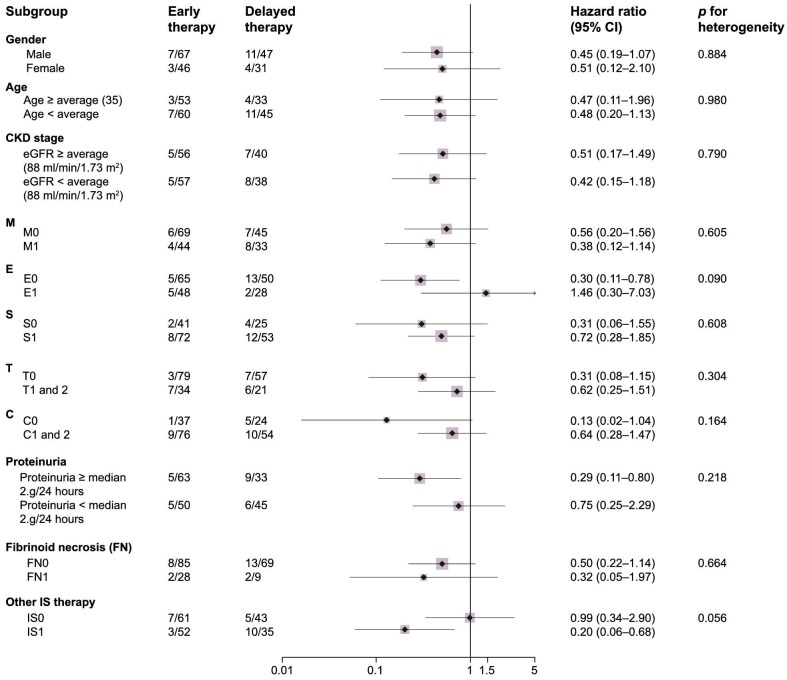
Primary outcome according to prespecified subgroups at baseline. IS: immunosuppressants; other IS therapy indicates cyclophosphamide, mycophenolate mofetil.

Subgroup analyses were conducted to examine the influence of baseline characteristics, including age, gender, initial eGFR, proteinuria levels at the time of kidney biopsy and MEST-C classification, pathology fibrinoid necrosis and whether combined with other immunosuppressants on the timing of steroid therapy. The results, summarized in Fig. 4, showed no evidence of heterogeneity for the primary outcome across prespecified subgroups at baseline.

### Sensitive analysis

Given that this was a retrospective study, we accounted for variability in the clinical and pathological baselines at the time patients initiated corticosteroid therapy. To address this, clinical baseline characteristics were adjusted to reflect parameters at the start of therapy, including age, 24-h urinary total protein, eGFR and MAP. Kaplan–Meier survival curve analysis showed a significant difference between the two groups (Log rank *P* = .014). After adjusting for gender, age, eGFR, UTP, MAP and Oxford Classification, the timing of steroid as an independent factor influencing long-term outcomes [*P* = .017; HR 0.27 (95% CI 0.10–0.80)] (Supplementary data, Table S1).

## DISCUSSION

This retrospective study focused on the timing of steroid therapy in the management of progressive IgAN and its impact on long-term outcomes. The findings showed that early initiation of corticosteroid treatment was associated with a significantly lower risk of renal failure over the 10-year follow-up period.

Randomized controlled trials, particularly the TESTING trial have demonstrated that steroid therapy is effective in managing progressive IgAN by reducing proteinuria and slowing the progression to renal failure [5]. However, the optimal timing for initiating steroid therapy in IgAN remains undefined. This uncertainty leaves clinicians with limited guidance on whether steroids should be prescribed immediately following a kidney biopsy or if delaying treatment initiation could yield better long-term renal outcomes.

In this study, we focused on analyzing the long-term prognosis of patients with IgAN who had an eGFR ≥50 mL/min/1.73 m^2^. This threshold aligns with KDIGO guidelines, which indicate that steroid therapy's risks often outweigh its benefits in patients with an eGFR <50 mL/min/1.73 m^2^ [15]. Using propensity score matching, we identified 191 matched IgAN patients. Kaplan–Meier survival analysis revealed that survival outcomes were significantly better in patients who initiated treatment in the early therapy group compared with those in delayed therapy group. Additionally, the timing of steroid administration was identified as an independent protective factor for long-term outcomes in IgAN, with an HR of 0.33 (95% CI 0.14–0.79, *P* = .013), although this result could overestimate the effect of timing of steroid in IgAN treatment because of the study design. For the secondary outcome, defined as a >30% or >40% decline in eGFR, the event rate was significantly lower in the early therapy group, consistent with the primary endpoint. Similarly, the average eGFR slope over the 10-year follow-up was more favorable in the early therapy group compared with the delayed therapy group. Regarding proteinuria, no significant difference was observed in time-averaged proteinuria within 1 year after corticosteroid therapy between the two groups.

Renal pathological findings are considered to reflect the activity level of nephritis, and the Oxford Classification (MEST-C) serves as a predictive tool for the progression to ESKD [17]. Patients with E and C lesions, as defined by the Oxford Classification, are thought to potentially benefit from steroid and immunosuppressant therapy [18]. However, robust evidence regarding the optimal timing of immunosuppressant treatment based on the Oxford Classification in IgAN is lacking. In the subsequent subgroup analysis, no heterogeneity was observed across variable groups, including age, gender, MEST-C classification, fibric necrosis lesions or the combination of immunosuppressant therapy. These findings suggest that the aforementioned factors did not influence the timing of steroid administration.

The use of systemic corticosteroid and the reduction of pathogenic IgA and IgA immune complex formation are emphasized in the KDIGO 2024 guidelines as key approaches to preventing nephron loss in the treatment of IgAN [8]. The benefits of early using steroid in IgA has not been debated. Evidence showed that mucosal-associated lymphoid tissue is responsible for the synthesis IgA1 molecular and further secreted pathogenic Gd-IgA1 and contribute to the development of IgAN [19, 20]. Early initiation of corticosteroid therapy can help prevent these pathogenic changes, preserving kidney function and preventing irreversible damage. In contrast, delayed corticosteroid treatment may result in a more established immune response, with significant IgA deposition and ongoing inflammation already present in the kidneys. In this IgAN cohort, six patients underwent repeated biopsies, as detailed in Supplementary data, Table S2. Four patients were in the early therapy group, and two patients were in the delayed therapy group. Among these, four patients achieved renal remission, and repeated biopsies were performed to explore pathological changes. The remaining two patients underwent re-biopsy due to worsening renal function. Notably, changes in IgA deposition were observed in two patients. Patient 1 (from the early therapy group) achieved renal remission, and follow-up biopsy showed reduced IgA deposition. In contrast, Patient 6 (from the delayed therapy group) experienced worsening renal function over 7 months without corticosteroid treatment, and biopsy revealed increased IgA deposition in the glomeruli. By this point, considerable renal damage may have already occurred, and corticosteroids may be less effective in reversing the damage. Therefore, early intervention is crucial to prevent further progression of kidney injury, particularly before fibrosis and irreversible glomerular damage become irreversible. However, more evidence is needed, and further detailed mechanistic studies and randomized controlled trials are required to confirm these findings.

This study has several limitations. First, as a retrospective analysis, the timing of steroid initiation (early or delayed) was not randomized. As a result, lifestyle modifications and the use of ARB/ACEi were unlikely to be fully balanced before the introduction of systemic corticosteroids. Additionally, follow-up durations varied significantly among patients, which may have influenced the outcomes. In this cohort, nearly 60% of patients initiated steroid therapy within 30 days, leaving the sample size treated after 30 days relatively small. This limited the statistical power to draw definitive conclusions. Furthermore, it remains challenging to determine whether initiating treatment at 30 days, 60 days or later would yield different outcomes or to identify the optimal timing for steroid therapy in IgAN.

## CONCLUSIONS

Our findings indicate that early corticosteroid therapy may help reduce renal decline and preserve long-term kidney function in IgAN patients requiring steroid treatment.

## Supplementary Material

sfaf076_Supplemental_File

## Data Availability

All data generated or analyzed during this study are included in this article and its online Supplementary data files. Further enquiries can be directed to the corresponding author.
