# Supplementary material for: Long-term evaluation of the timing of corticosteroid therapy in an IgA nephropathy cohort
Source: Clin Kidney J. 2025 Mar 17;18(5):sfaf076. doi: 10.1093/ckj/sfaf076 (PMC12044332; doi:10.1093/ckj/sfaf076)
Supplement: sfaf076_Supplemental_File [file sfaf076_supplemental_file.docx]

**Supplementary Materials**

**
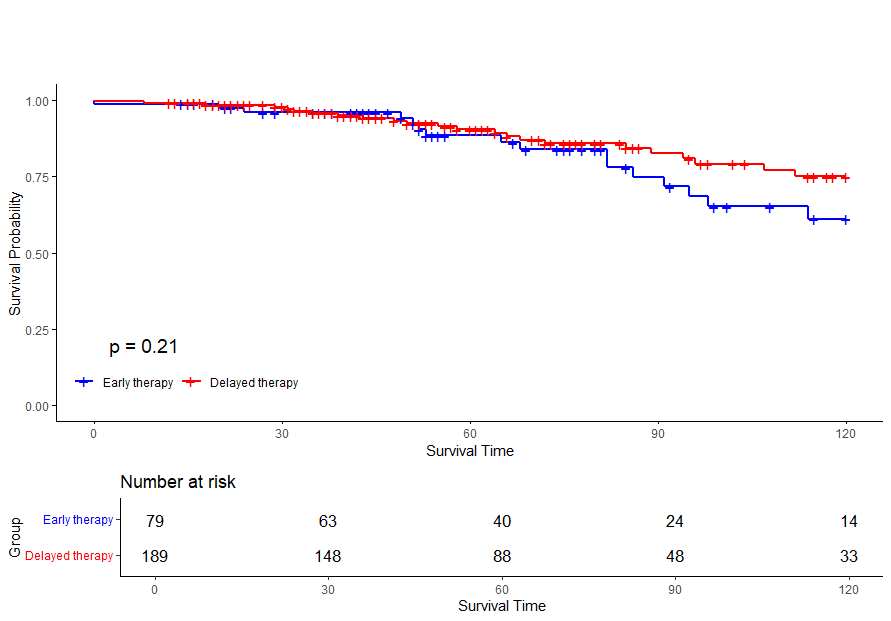
Supplementary Fig 1. Kaplan-Meier analysis of the primary outcome in 268 IgAN patients**

| **Supplementary Table 1. Cox Regression Analysis of Factors Associated with Composite Renal Outcomes (Baseline Characteristics Replaced with Pre-Corticosteroid Values)** | | | | | |
| --- | --- | --- | --- | --- | --- |
|  | **Univariate analysis** | |  | **Multivariate analysis*** | |
|  | HR(95%) | *p* value |  | HR(95%) | *p* value |
| **Group** |  |  |  |  |  |
| Delayed Therapy | reference |  |  | reference |  |
| Early Therapy | 0.29 (0.10, 0.83) | 0.021 |  | 0.27 (0.10, 0.80) | 0.017 |
| **Age (years)** | 0.99 (0.95, 1.03) | 0.508 |  |  |  |
| **GENDER M/F** | 0.79 (0.29, 2.14) | 0.641 |  |  |  |
| **GFR.EPI (mL/min/1.73 m2)** | 0.98(0.96, 1.01) | 0.168 |  |  |  |
| **Proteinuria UTP (g/day)** | 1.153 (0.95, 1.39) | 0.142 |  |  |  |
| **MAP** | 1.02(0.98, 1.06) | 0.286 |  |  |  |
| **Oxford classification** |  |  |  |  |  |
| **Mesangial hypercellularity** |  |  |  |  |  |
| (M0/M1) | 1.346 (0.51, 3.54) | 0.547 |  |  |  |
| **Endocapillary hypercellularity** |  |  |  |  |  |
| (E0/E1) | 0.43 (0.12, 1.49) | 0.181 |  |  |  |
| **Segmental glomerulosclerosis** |  |  |  |  |  |
| (S0/S1) | 1.42 (0.50, 4.03) | 0.512 |  |  |  |
| **Tubular atrophy/interstitial fibrosis** |  |  |  |  |  |
| (T0/1) | 2.87 (1.04, 7.93) | 0.042 |  | 3.16 (1.14, 8.77) | 0.027 |
| (T1/2) | 4.26 (0.90, 20.11) | 0.067 |  | 3.82 (0.81, 18.11) | 0.091 |
| **Crescents** |  |  |  |  |  |
| (C0/1) | 1.06(0.37, 3.05) | 0.920 |  |  |  |
| (C1/2) | 0.53 (0.06, 4.51) | 0.558 |  |  |  |

| **Supplementary Table 2.**  **Comparison of the Details of Two Renal Biopsies** | | | | | | | | |  |
| --- | --- | --- | --- | --- | --- | --- | --- | --- | --- |
|  | **Age/Gender** | **Early Therapy** | **Reasons for repeated biopsy** | **Biopsies Interval**  **(months)** | **Changes** | | | |  |
|  |  |  |  |  | **Serum IgA^*^** | **IgA Deposits** | **eGFR**  **(ml/min/1.73m^2^)** | **24-hour proteinuria(g/24h)/**  **Hematuria(HPF^§^)** |  |
| **Patient 1** | **16/Male** | **Yes** | **Renal remission,**  **pathological exploration** | **3** | **①2.77**  **②3.09** | **①IgA++++**  **②IgA+++** | **①91**  **②87** | **①4.35/100-130**  **②0.31/2-5** |  |
| **Patient 2** | **20/Male** | **Yes** | **Renal remission,**  **pathological exploration** | **42** | **①3.19②3.20** | **①IgA++**  **②IgA++** | **①127**  **②93** | **①4.27/40-50**  **②0.75/10-15** |  |
| **Patient 3** | **38/Female** | **No** | **Renal remission,**  **pathological exploration** | **18** | **①4.32②4.05** | **①IgA+++**  **②IgA+++** | **①113**  **②78** | **①5.25/30-40**  **②0.29/5-10** |  |
| **Patient 4** | **45/Male** | **Yes** | **Renal remission,**  **pathological exploration** | **10** | **①2.88②2.74** | **①IgA+++**  **②IgA+++** | **①70**  **②67** | **①4.86/80-100**  **②0.23/10-20** |  |
| **Patient 5** | **17/Male** | **Yes** | **Worse in renal function** | **32** | **①1.67②2.03** | **①IgA+++**  **②IgA+++** | **①61**  **②48** | **①16.40/150-200**  **②1.87/3-12** |  |
| **Patient 6** | **49/Female** | **No** | **Worse in renal function** | **7** | **①4.07②3.66** | **①IgA++**  **②IgA+++** | **①72**  **②19** | **①1.90/40-50**  **②3.44/100-150** |  |

*** ①The first kidney biopsy ②The second kidney biopsy**

**§ HPF : per high power field (HPF)**
